# Allogeneic Mesenchymal Stem Cell Treatment Induces Specific Alloantibodies in Horses

**DOI:** 10.1155/2016/5830103

**Published:** 2016-08-28

**Authors:** Sean D. Owens, Amir Kol, Naomi J. Walker, Dori L. Borjesson

**Affiliations:** Department of Pathology, Microbiology, Immunology, School of Veterinary Medicine, University of California, Davis, CA 95616, USA

## Abstract

*Background.* It is unknown whether horses that receive allogeneic mesenchymal stem cells (MSCs) injections develop specific humoral immune response. Our goal was to develop and validate a flow cytometric MSC crossmatch procedure and to determine if horses that received allogeneic MSCs in a clinical setting developed measurable antibodies following MSC administration.* Methods.* Serum was collected from a total of 19 horses enrolled in 3 different research projects. Horses in the 3 studies all received unmatched allogeneic MSCs. Bone marrow (BM) or adipose tissue derived MSCs (ad-MSCs) were administered via intravenous, intra-arterial, intratendon, or intraocular routes. Anti-MSCs and anti-bovine serum albumin antibodies were detected via flow cytometry and ELISA, respectively.* Results.* Overall, anti-MSC antibodies were detected in 37% of the horses. The majority of horses (89%) were positive for anti-bovine serum albumin (BSA) antibodies prior to and after MSC injection. Finally, there was no correlation between the amount of anti-BSA antibody and the development of anti-MSC antibodies.* Conclusion.* Anti allo-MSC antibody development was common; however, the significance of these antibodies is unknown. There was no correlation between either the presence or absence of antibodies and the percent antibody binding to MSCs and any adverse reaction to a MSC injection.

## 1. Introduction

Allogeneic mesenchymal stem cells (MSCs) are being investigated in both human and equine studies. Intravenous (IV) infusion of allogeneic MSCs appears to be safe, and no significant adverse effects have been reported [1, 2]. In comparison to autologous MSCs, allogeneic MSCs offer the advantage of being a thoroughly characterized cellular product that is immediately available to treat patients with an acute injury without the requisite delay associated with the culture and expansion of autologous MSCs [3]. Further, allogeneic MSC use is the current standard of care in the majority of human clinical trials [3, 4]. Unlike blood transfusion or solid organ transplantation, where tissue typing is required to prevent blood/graft rejection, allogeneic MSC use without any tissue typing or pretransfusion testing is considered possible given that MSCs are immune evasive and their expression of human leukocyte antigen (HLA) class II molecules is negligible [3–5].

While allogeneic MSCs were previously considered to be immune privileged and to not induce an alloimmune reaction [6], it is now recognized that low levels of cellular as well as humoral alloimmunity can be identified in humans and horses that have been treated with allogeneic MSC [3, 7, 8]. However, many basic questions concerning the hemocompatibility of MSCs [5] and their fate after intralesional and systemic infusion remain unanswered [6, 9]. Although alloimmune recognition of MSCs may injure infused MSCs, decrease their survival time, and impede clinical outcome, there is currently no data to support this hypothesis.

MSC route of administration, tissue source, final formulation, and the dosage frequency are variables that have the potential to affect the development of anti-MSC alloantibodies. Intradermal injection of BM-MSCs induced the formation of specific anti-MSC antibody in 6 healthy horses in one study [8]. MSCs, regardless of tissue origin, are of inherently low immunogenicity in vitro, and studies with human and animal MSCs (dogs and pigs) suggest that the use of allogeneic, unmatched MSCs is feasible. However, MSCs do not completely evade immune surveillance and have been shown to induce allograft responses in immunocompetent rhesus macaques [10]. However, little is known with regard to equine antibody development as a result of cell source, dosage, or frequency of administration in diseased horses.

The objectives of this study were to (1) develop and validate an equine specific flow cytometric MSC crossmatch procedure and (2) to determine if horses that received allogeneic MSCs developed measurable antibodies following allogeneic administration of MSCs from different tissue sources via different routes of administration. We hypothesized that horses would develop measurable antibodies to allogeneic MSCs regardless of route of administration, frequency of administration, or MSC source.

## 2. Materials and Methods

### 2.1. Flow Cytometric Crossmatch Assay

We modified a flow cytometric MSC crossmatch procedure that was developed for use with human patients to identify transplant associated anti-MSC alloantibodies ([6] and Dr. Edwin Horwitz, Immunogenetics Laboratory, the Children's Hospital of Philadelphia, personal communication). Cryopreserved MSCs were thawed exactly as previously described in a 37°C water bath [12, 13]. Thawed MSCs were transferred into 15 mL polypropylene tubes (Falcon, BD Biosciences, Franklin Lakes, NJ) with warm Dulbecco's Phosphate Buffered Saline (DPBS, Gibco, Invitrogen, Carlsbad, CA) and centrifuged gently (110*g*, 10 min). The supernatant was removed and the cells were resuspended in DPBS for a 30-minute rest at room temperature (RT). Cells were washed (410*g*, 10 min) and then incubated in blocking solution (2% normal goat serum (Jackson ImmunoResearch Inc., West Grove, PA)) in DPBS while rocking at RT for 30 min. Cells were pelleted (410*g*, 10 min), the supernatant was removed, and cells were washed 3 times with DPBS. Horse sera (MSC recipient serum) was heat inactivated and diluted 1 : 1 with 12.5% normal horse serum (HyClone, Logan, UT) in DPBS. 200 *µ*L of this diluted test sera was added to each tube; the sample was vortexed briefly and permitted to incubate at RT for 30 min. After incubation, MSCs were washed 3 times (1000*g*, 5 min) with flow buffer (0.5% bovine serum albumin and 5 mM EDTA in DPBS). After washing, 100 *µ*L of the secondary antibody (rabbit polyclonal antibody to equine IgG-FITC, diluted 1 : 200, Abcam, Cambridge, MA) was added to the cell pellet, mixed, and permitted to incubate for 20 min at RT in the dark. Cells were washed 3 times (1000*g*, 5 min), resuspended in flow buffer, and analyzed on a flow cytometer (Cytomics FC 500, Beckman Coulter, Brea, CA). Flow cytometry data were analyzed using FlowJo flow cytometry software (Tree Star Inc., Ashland, OR, USA).

Samples included a positive erythrocyte (RBC) control, 3 negative controls, and the test condition. The positive control was developed to confirm that the rabbit polyclonal anti-equine IgG-FITC antibody appropriately detected cells bound with antibody. For the positive control we mixed RBCs from Aa+ horses with serum from Aa− horses with a confirmed anti-Aa alloantibody. While equine red cell antigen alloantibodies are both agglutinating and hemolytic, anti-Aa antibodies are predominantly agglutinating [14]. In brief, blood was collected from an Aa+ horse via jugular venipuncture in vacutainer tubes containing Acid Citrate Dextrose (BD Biosciences), centrifuged at 1000*g*  × 1 min, and the plasma and buffy coat were removed. Packed RBCs were diluted with DPBS and 1 × 10^6^ RBCs were mixed from serum from an Aa− horse (diluted 1 : 3 with 12.5% normal horse serum in DPBS) and incubated at RT for 20 min. RBCs were washed 2x with flow buffer (1000*g*, 1 min) after which the antibody to equine IgG (IgG-FITC, diluted 1 : 200, Abcam) was added. Cells were vortexed and then incubated for 20 min at RT in the dark. Cells were washed as above and samples were read using the flow cytometer. Negative control samples consisted of (1) RBCs from an Aa− horse mixed with serum from an Aa− horse, (2) MSCs with just the secondary antibody (no primary antibody (serum) control), and (3) recipient horse serum mixed with “irrelevant” MSCs (MSCs from a different donor to which the recipient had not been exposed). This essentially served as a background binding control to rule out nonspecific binding of serum to MSCs. A threshold (i.e., negative) cut-off was determined based on the binding of serum to irrelevant MSC. Anti-MSC antibodies were considered positive if the serum bound more than 3% of the tested MSCs over the upper range of the background binding to accommodate for the variation in serum binding to MSCs at baseline (prior to MSC administration).

### 2.2. Detection of Anti-BSA Antibodies

An ELISA was adapted from Gershwin et al. [15] to detect antibodies directed against the primary bovine protein in fetal bovine serum (FBS) and bovine serum albumin (BSA). Briefly, 96-well ELISA plates (Nunc-Immuno MaxiSorp, Thermo Fisher Scientific, Rochester NY) were coated with 100 *µ*L BSA (1 *µ*g, Fraction V, Fisher) in a carbonate-bicarbonate buffer (63.5 mM carbonate, pH 9.5) overnight at 4°C. To block, 100 *µ*L of 1% rabbit serum albumin (Sigma, St. Louis, MO) in DPBS was added to each well and incubated at 37°C for 1 hr. Wells were washed with DPBS + 0.1% Tween 20 (wash buffer, EMD Chemicals, San Diego, CA) once for 10 minutes and then 6 times briefly. 100 *µ*L of test serum (diluted 1/1000 in wash buffer) was added to each well. Each sample was plated in triplicate. Known negative and high positive samples were run as assay controls. Plates were incubated at 37°C for 1 hr and washed as above and then 100 *µ*L of rabbit anti-equine IgG H&L-HRP (diluted 1 : 200,000, Abcam) was added to each well. Plates were incubated at 37°C for 1 hr and washed as above, 100 *µ*L of TMB Peroxidase Substrate (KPL, Gaithersburg, MD) was added to each well, and plates were incubated at RT in the dark. The colorimetric reaction was stopped with 100 *µ*L of 2 N H_2_SO_4_ and plates were read at 450–540 on a Synergy HT Multi-Mode microplate reader with Gen5 software (BioTek, Winooski, VT). Fold increase in color relative to the negative control was determined for each sample.

### 2.3. MSC Donor Horses and MSC Culture

Fat and BM were collected from healthy horses housed at the Center for Equine Health (CEH) at the University of California, Davis (UCD), exactly as previously [1, 16]. These horses are routinely vaccinated, dewormed, and screened for viral pathogens. MSCs were isolated, cultured, and characterized exactly as previously described [11, 16]. Briefly, bone marrow mononuclear cells and adipose tissue stromal vascular fraction were cultured in Dulbecco's Modified Eagle Medium (Gibco) supplemented with 10% FBS (Atlanta Biologicals, Flowery Branch, GA) and 1% Penicillin-Streptomycin (Gibco). Cells were passaged at ~70% confluence. MSCs were characterized by stromal cell morphology, plastic adherence, and surface protein expression (MHC-I, CD90, CD44, and CD29 positive and MHC II, CD86, and F6B (an equine pan-leukocyte marker) negative) [17].

### 2.4. Study Horses

Serum was collected from a total of 19 horses enrolled in 3 different research projects (recipient horses) [1, 18]. All horses were healthy, adult horses housed at the CEH at UCD. All 3 studies were performed according to approved institutional animal care and use committee protocols (UCD). Serum from all horses was collected on 2 occasions: prior to MSC injection and 2 weeks after the final MSC injection. Serum was aliquoted and frozen at −80°C until analysis.

All horses in the 3 studies received fully unmatched allogeneic MSCs (see Table 1) [1, 18]. In the first study (a.k.a., tendon study), horses received 4 injections of 25–80 million BM-MSCs per injection (total dose: ~100–320 million BM-MSCs/horse) [18]. MSCs were administered via intravenous regional limb perfusion, via intra-arterial regional limb perfusion, and via direct injection into an experimentally induced tendon lesion [19]. For this study, cells from 2 different donor horses were used. For any given recipient, repeated injections were all from the same donor horse. In the second study (a.k.a., eye study), horses received 2 intravitreal injections of fat-derived MSCs (Ad-MSCs, Borjesson, unpublished data). The first injection contained 25 million MSCs and the second injection contained 50 million MSCs (total dose was 75 million MSCs/horse). For this study, cells from 2 different donor horses were used. For any given recipient, repeated injections were all from the same donor horse. In the third study (a.k.a., IV study), horses received 3 intravenous injections of 25 million Ad-MSCs or BM-MSCs per injection (total dose was 75 million MSCs/horse) [1]. For this study, cells from 5 different donor horses were used. For any given recipient, repeated injections were all from the same donor horse.

### 2.5. Statistical Analysis

A Student's *t*-test was used to compare background serum binding to BM-MSCs and Ad-MSCs.

## 3. Results

### 3.1. MSC Crossmatch Assay Development

**Figure 1:**
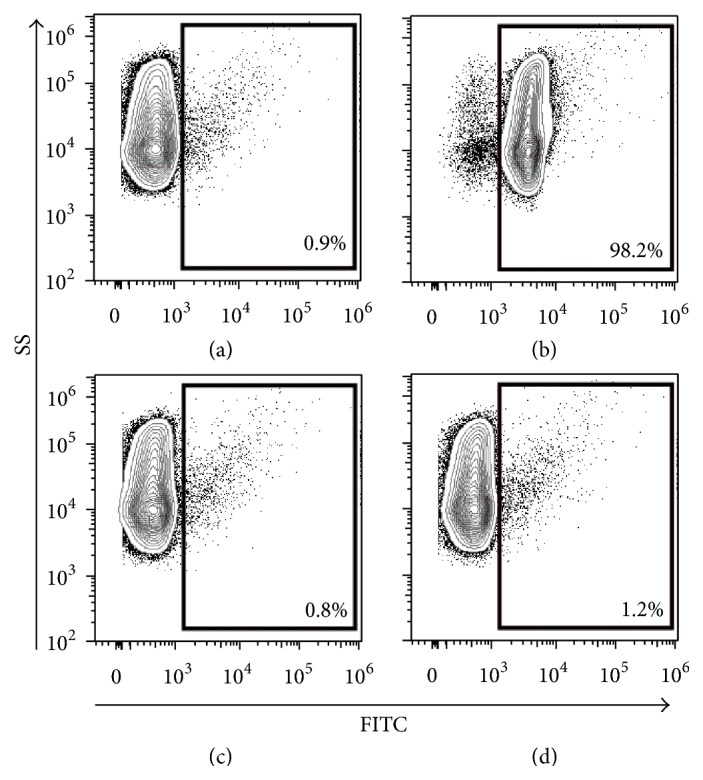
RBC crossmatch procedure to validate rabbit anti-equine polyclonal IgG antibody binding to equine cells. (a) Aa+ RBCs mixed with secondary antibody (no serum (primary antibody) control), (b) Aa+ RBCs mixed with serum from an Aa− horse that contains alloantibodies to Aa+ RBCs, (c) Aa− RBCs mixed with secondary antibody (no serum (primary antibody) control), and (d) Aa− RBCs mixed with serum from an Aa− horse that does not contain antibodies to Aa− RBCs.

The assay was developed using serial dilutions of primary antibody (recipient horse serum) titrated with serial dilutions of secondary antibody (rabbit anti-equine IgG-FITC). Multiple blocking reagents and blocking times were evaluated to minimize background serum binding to MSCs (2% normal goat serum provided the most complete block of nonspecific serum binding to cells). The sensitivity and specificity of a commercially available rabbit anti-equine IgG antibody were confirmed by incubating equine serum with or without naturally occurring alloantibodies to Aa+ RBCs with Aa+ and Aa− RBCs, respectively, as positive and negative controls, respectively (Figure 1). In order to determine nonspecific binding of equine serum (from untreated animals) to equine MSC, forty-two serum samples, collected from horses prior to any exposure to MSCs, were used to determine background binding of serum to both BM-MSCs (*n* = 22) and Ad-MSCs (*n* = 20) (Table 2). Background serum binding to BM-MSCs was significantly higher than background serum binding to Ad-MSCs (*p* < 0.001). As such, for study samples, the final percent positive binding is reported as the total binding of serum antibodies to administered MSCs minus the background binding to “irrelevant” MSCs that were not administered to the horse from which the tested serum was obtained. A serum sample was considered “positive” for anti-MSC antibodies if the serum bound to >3% of the tested MSCs over background binding (see details in Materials and Methods).

### 3.2. Crossmatch Results for Study Horses

**Figure 2:**
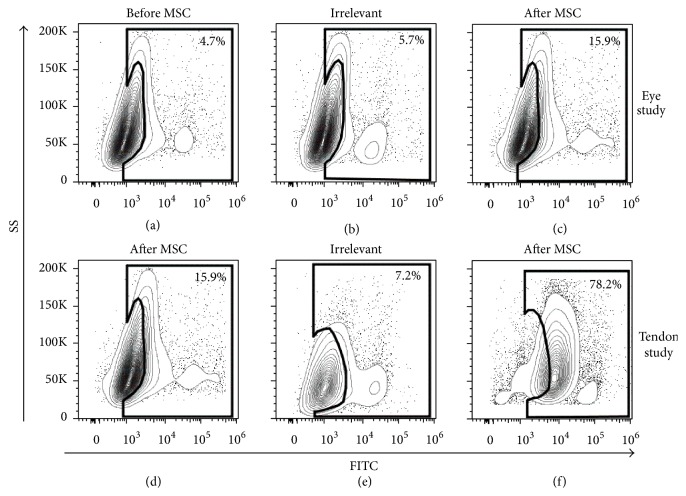
Representative flow cytometric contour plots of serum antibody binding to equine MSCs (eye study, (a)–(c); tendon study, (d)–(f)). (a) Percent positive antibody binding to MSCs prior to any in vivo MSC administration (background binding). (b) Percent positive antibody binding to irrelevant (nondonor) MSCs after in vivo MSC administration (nonspecific binding). (c) Percent positive antibody binding to donor MSCs 2 weeks after final in vivo MSC administration. (d) Percent positive antibody binding to MSCs prior to any in vivo MSC administration (background binding). (e) Percent positive antibody binding to irrelevant (nondonor) MSCs after in vivo MSC administration (nonspecific binding). (f) Percent positive antibody binding to donor MSCs 2 weeks after final in vivo MSC administration.

Nineteen horses had paired pre- and post-MSC administration serum samples (*n* = 4, tendon study; *n* = 5, eye study; *n* = 10, IV study; see Table 1). These results are summarized in Table 3. Forty percent (2/5) of the horses in the eye study developed a mild (<5% binding) and specific antibody response to the Ad-MSCs which they received (Figures 2(a)–2(c)). Ten percent (1/10) of the horses in the IV study developed a mild and specific antibody response to the BM-MSCs which they received. All 4 horses in the tendon study developed marked and specific (>15% binding) antibodies that bound to the BM-MSCs which they received as compared to pre-MSC administration serum samples in which no binding was observed (Table 2; Figures 2(d)–2(f)). Overall, antibodies were detected in 7/19 horses (37% of the study horses), 5 of which received BM-MSCs and 2 of which received Ad-MSCs.

Although the specific determination of alloantibody duration and persistence were not objectives for any of the 3 studies, after the detection of alloantibodies in many horses, we went back to the study population to collect additional serum samples, as available. For example, for the only horse enrolled in the IV study that developed measurable antibodies, multiple serum samples were available over the course of the study. For this animal, antibodies were not present at day 28 after MSC injection but developed and peaked at day 35 after MSC injection (at which time the horse had received 3 MSC injections). However, antibodies were no longer detectable at day 84 after MSC injection. For 4 additional horses for which samples were available (2, eye study, and 2, tendon study), antibodies developed by day 25 after MSC injection (at which time 2 MSC injections had been received). The percent binding increased in one horse (tendon study, day 420 after injection) and decreased in the remaining 3 horses (by day 42, day 585, and day 600 after MSC injection). These data suggest that antibodies to MSCs may develop 3-4 weeks after MSC injection and that antibodies mostly diminish over time; however, they may persist.

### 3.3. Anti-BSA Antibodies in Study Horses

**Figure 3:**
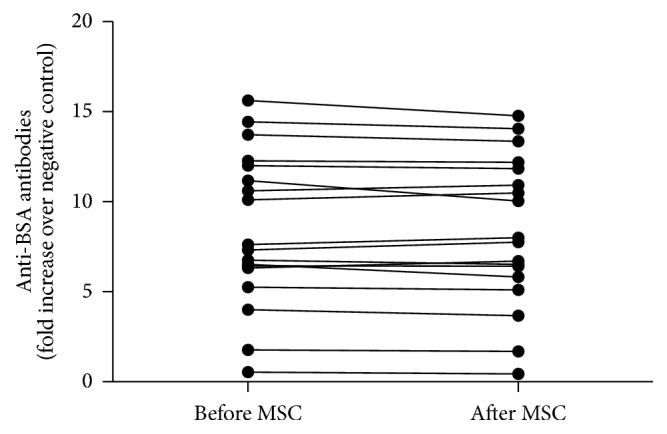
Anti-BSA antibodies in horse prior to and after MSC administration. Sera from 18 horses were available for anti-BSA antibodies determination via ELISA. Each dot represents serum from a horse prior to MSC administration and sera from the same horse after MSC administration. Before and after dots from each horse are connected with a line. Horses with no measurable anti-BSA antibodies served as negative controls and anti-BSA titer is presented as fold increase over these negative controls. While 16 of the 18 horses (89%) had positive anti-BSA antibodies titer prior to MSC administration, none of the horses developed higher antibodies titer after MSC administration. Moreover, the 2 horses that did not have measurable anti-BSA antibodies prior to MSC administration did not seroconvert after MSC administration.

Eighteen of the 19 study horses had both before and after serum samples available for the anti-BSA ELISA assay. Sixteen of these 18 research horses housed at the CEH had high preexisting anti-BSA antibody titers ranging from 4 to 16 times higher than the negative control sample (horses with no detectable anti-BSA antibodies) [15]. Two horses were essentially negative for anti-BSA antibodies (defined as a fold increase <2 compared to negative control samples). None of the 18 horses showed any change in anti-BSA antibodies when sampled prior to MSC therapy or up to 2 weeks after the last injection (Figure 3). There was also no correlation between high anti-BSA titers and the presence of detectable anti-MSC titers (all 7 horses that developed measurable anti-MSC antibodies had measurable anti-BSA titers; range: 7.3–15 times compared to that that did not change over time).

## 4. Discussion

In most acute clinical diseases or syndromes, allogeneic MSCs would be the only option for timely treatment using cell-based therapies. Given that MSCs are of low immunogenicity and are believed to be immune evasive [3, 9, 20], the possibility of “universal donor” MSCs for clinical use is worth exploring. We propose that the use of a stem cell crossmatch technique, in much the same way that a crossmatch procedure is used for RBC transfusions, would provide important information as to the pretransfusion compatibility of MSC therapies. Towards that end, in this study, we developed a flow cytometric based MSC crossmatch procedure and used it to determine that equine patients, following exposure to allogeneic MSCs, may develop anti-MSC alloantibodies. These findings are consistent with recent manuscripts that reported the formation of anti-allogeneic MSC antibodies following intradermal allogeneic MSC injection in 6 healthy horses [8]. In our work, antibody development was fairly common; however, the significance of these antibodies is unknown. There was no correlation between either the presence or absence of antibodies or the percent antibody binding to MSCs and any adverse reaction to MSC injection. Similar findings have been described in rats [21] and in baboons following multiple, high-dose (5 × 10^6^ MSCs/kg body weight) administration of allogeneic MSCs [22].

Antibodies developed to the greatest degree (both in terms of the highest number of horses and the highest antibody binding) in the horses involved in the tendon study. There are a number of potential reasons for strong antibody responses in these horses. These horses received the highest total number of MSCs, the highest number of injections, and the most variation in routes of administration. These horses also had an experimentally induced lesion (horses in the other two studies were healthy with no known lesions). The specificity, target epitope, or cross-reactivity of these alloantibodies is unknown.

Consistent with previous findings, it may be hypothesized that these alloantibodies may be directed at blood group antigens present on the surface of MSCs [8]. While Sundin and colleagues [6] have shown that human MSCs do not express blood group antigens, recently Pezzanite et al. [8] showed that horses that were negative for equine leukocyte antigen-A2 (ELA-A2) that received MSCs from ELA-A2 positive donors developed ELA-A2 specific alloantibodies, suggesting that MSCs express ELA-A2. The specific MSC antigens that are most immunogenic have not yet been determined.

An additional consideration for the development of alloantibodies would be the fact that these alloantibodies developed in response to exposure to FBS [6]. MSCs are frequently cultured in medium supplemented with FBS, and it has been reported that MSCs both internalize and have FBS components present on the cell surface. Sensitization to xenogenic serum proteins used to prepare vaccines or other cell-based products has been reported in several species [15, 23–25]. Annual and/or semiannual vaccination of horses with multiple vaccines (created in FBS-containing media) is common in the equine industry and thus horses typically develop strong antibodies to BSA (the major component of FBS). The measurement of anti-BSA antibodies is also frequently measured in human clinical trials [26]. In one study, the development of anti-BSA antibodies was correlated to treatment failure [26]. MSCs administered to our client-owned patients are commonly cultured in 10% FBS and thus we wanted to distinguish between an antibody response that may be directed at the MSCs and an antibody response directed towards a primary xenoantigen in which the cells are cultured. Rat MSCs grown in FBS-supplemented medium gave rise to a humoral response after recurrent administrations in immunocompetent animals [27]. We found that although most of all our study horses had high preexisting titers to BSA, the primary component of FBS, multiple MSC injections did not result in any measurable or significant change in antibody titer over time. As such, the anti-MSC antibody measured in this study was not directed specifically towards bovine xenoproteins.

There are several limitations to this retrospective study. First, this study was a retrospective study using samples collected from healthy horses enrolled in 3 distinct in vivo studies in which horses received allogeneic MSC from different tissue sources via different routes and in different concentrations. Although that makes the data more difficult to directly compare, it does give strong sampling of the potential responses in the many small studies that are done in the horse. Second, we did not attempt to identify the alloantibody with regard to immunoglobulin class (i.e., IgM, versus IgG, etc.) or the target epitope(s) of the identified alloantibodies nor did we determine if the alloantibodies were cytotoxic. Third, each of the three research groups contained a small sample size. And finally, we did not determine whether antibodies developed to autologous MSCs.

*In conclusion*, there is increasing evidence to suggest that allogeneic MSCs may elicit an immune response in the recipient, and our results add to the growing body of evidence which demonstrates that allogeneic donor MSCs are not fully immune privileged in an immunocompetent recipient. Such results may serve to reduce the therapeutic potential of allogeneic MSCs; however, understanding the exact nature of the immune response against allogeneic MSCs might help in developing strategies to optimize the therapeutic use of allogeneic MSCs and minimize negative immune effects or reduce the risk of possible rejection. Future work evaluating the prospective use of this crossmatch procedure in clinical and research patients receiving both allogeneic and autologous MSCs may prove beneficial in more fully understanding the clinical importance of alloantibody production.

## Figures and Tables

**Table 1:** In vivo study designs.

Study	Horses number	MSC tissue source	Route of injection	MSC dosage (×10^6^)	Number of injections	Reference
Tendon	4	Bone marrow	Intravenous, intra-arterial, and intralesional	25–80	4	[11]

Eye	5	Adipose tissue	Intravitreal	25 and 50	2	[Borjesson, unpublished]

IV	5	Adipose tissue	Intravenous	25	3	[1]
	5	Bone marrow	Intravenous	25	3	[1]

**Table 2:** The percentage of background serum antibody binding to equine BM-MSCs and Ad-MSCs.

	Before samples	
	*BM-MSCs*	*Ad-MSCs*
*n*	22	20
Minimum	3.37%	2.40%
Maximum	13.73%	7.52%
Median	**7.69%** ^*∗∗*^	**4.06%**

^*∗∗*^ *p* < 0.01.

**Table 3:** The percentage of antibody binding to equine MSCs prior to and after MSC administration.

	Study horses							
	*Tendon*		*Eye*		*IV*			
*n*	4		5		5		5	
MSC source	BM-MSC		Ad-MSC		BM-MSC		Ad-MSC	
	Before (%)	After (%)	Before (%)	After (%)	Before (%)	After (%)	Before (%)	After (%)
Minimum	0.00	19.68	0.00	0.00	0.00	0.00	0.00	0.00
Maximum	0.00	48.56	1.38	12.00	4.96	13.42	1.21	6.70
Median	*0.00*	***41.68*** ^*∗∗*^	*0.38*	***0.00***	*0.21*	***0.00***	*0.24*	***1.09***
Average	*0.00*	***37.90***	*0.53*	***4.53***	*1.46*	***3.25***	*0.43*	***1.93***

^*∗∗*^Significant difference between % antibody binding before MSC administration and % antibody binding after MSC administration.

## Abbreviations

Ad: Adipose
BM: Bone marrow
BSA: Bovine serum albumin
CEH: Center for Equine Health
DPBS: Dulbecco's Phosphate Buffered Saline
ELA: Equine leukocyte antigen
FBS: Fetal bovine serum
HLA: Human leukocyte antigen
IV: Intravenous
MSC: Mesenchymal stem cells
RBC: Red blood cells
RT: Room temperature
UC: University of California.
